# Healthcare Workers’ Emotions, Perceived Stressors, and Coping Strategies at Workplace during the COVID-19 Pandemic in Palestine

**DOI:** 10.3390/ijerph191911966

**Published:** 2022-09-22

**Authors:** Muna Ahmead, Nuha El Sharif, Samer Asad

**Affiliations:** 1 Faculty of Public Health, AlQuds University, Jerusalem P.O. Box 51000, Palestine; 2 Ministry of Health, Ramallah 4284, Palestine

**Keywords:** COVID-19, health professional, nurse, doctor, motivation, pandemic, coping strategies, workplace

## Abstract

Evidence about the impact of the COVID-19 pandemic on the mental health of Palestinian healthcare professionals is lacking and has been disregarded during the COVID-19 pandemic. This study aims to describe Palestinian healthcare workers (HCWs) emotions and factors causing stress, and factors used to reduce the stress experienced at the workplace and to examine the relationships between HCWs level of stress and their coping strategies and motivational factors during the COVID-19 pandemic. A self-reported online survey was completed by 506 doctors and nurses working in healthcare facilities that provide COVID-19 care. Descriptive statistics, bivariate, and multivariate regression models were developed to adjust for the association between HCWs coping and motivation factors with HCWs’ perceived stressors. The findings showed that 24.1% of the participants had a high-stress level, and 25.7% had a low level of stress. The participant’s main coping strategy was avoidance and the key emotional factor was the ethical and professional obligation to continue working. Additionally, a major cause of their stress was their personal safety and having the COVID-19 infection. Findings showed a positive association between stress and the younger age of participants, with physicians being less stressed than nurses. In addition, receiving no training on the treatment of COVID-19 was strongly associated with stress levels. Furthermore, there was a significant positive correlation between stress scoring and coping strategies scoring but not with motivation score. In conclusion, this study shows that Palestinian healthcare workers experienced emotional turmoil during the COVID-19 pandemic. These results indicate the necessity of providing supervision, psychological counseling and intervention to professional healthcare workers who work directly with COVID-19 patients in health settings during the current pandemic or in the event of future outbreaks. Policymakers and managers must also conduct training and provide interventions on how to cope with stress in pandemics, in order to assist HCWs in developing their adaptive coping strategies and increase their motivation

## 1. Introduction

The coronavirus disease (COVID-19) pandemic began in late December 2019, posing a challenge to psychological resilience worldwide and, in particular, to healthcare professionals. It has caused millions of cases and deaths globally [1,2,3] and had a tremendous impact on healthcare systems and healthcare workers’ mental health [4]. At the beginning of the coronavirus pandemic, hospitals had limited availability of resources, in terms of empty beds, trained staff, personal protective equipment, and treatment protocols [5]. These factors affected professional health workers who were unprepared for a pandemic [6]. Feelings of uncertainty, isolation, and loneliness increased, in addition to a sense of stigma [7,8]. Many studies reported that healthcare teams working in COVID-19 units and hospitals faced enormous pressure due to the risk of infection, inadequate protection from contamination, the wearing of personal protective equipment (PPE) for long working hours [6,9], overwork, frustration, discrimination, isolation, a lack of contact with families, exhaustion, and dealing with patients with negative emotions [10].

During COVID-19, several studies reported that healthcare teams suffered from stress, anxiety, symptoms of depression, insomnia, denial, anger, and fear [7,11], in addition to a lack of training in infection control and limited access to psychological intervention and support [12]. Khanal et al. (2020), found that 41.9% of health workers had symptoms of anxiety, 37.5% suffered from depression, and 33.9% had insomnia. Nurses experienced more anxiety symptoms than other health workers. The study concluded that participants without adequate protective equipment in their health settings were more at risk of developing mental health problems [4]. Furthermore, a study by Giusti et al. (2020), found that, of 330 health professionals, 235 of them (71.2%) had clinical anxiety, 88 (26.8%) had clinical depression, 113 (34.3%) suffered from stress, and 121 (36.7%) suffered from post-traumatic stress [10].

During pandemic situations, there are coping strategies that may help healthcare professionals cope with the stressors associated with infectious disease outbreaks, including clear instructions, adequate training, and support at the workplace, as well as social, religious, and family support [13,14].

The act of coping with stress motivates individuals to engage in certain coping behaviors [15]. A person’s motivation is the factor that leads to their satisfaction or dissatisfaction prior to making a decision, according to Herzberg (1968) [16]. Therefore, it may influence coping strategies by regulating the appraisal or experience of stress. During the first wave of the COVID-19 pandemic, HCWs reported high levels of stress and emotional trauma, which affected their motivation and coping strategies [17]. A study suggested that nurses working in a COVID-19 ward should receive psychological support in the form of “timely psychological assistance”, as well as training in coping strategies. Researchers concluded that nurses might benefit from training in regulating their emotions and developing a better coping strategy in situations that demand resilience to combat the pandemic [18].

In Palestine, the first cases of COVID-19 were reported in early March 2020 in the Bethlehem Governorate and the outbreak quickly overwhelmed the health services. Despite the nationwide lockdown imposed on the 5th of March, the number of cases and deaths accelerated. By January 2021, the Ministry of Health had recorded 183,365 cases and 2072 deaths in the country [19]. However, evidence about the impact of the COVID-19 pandemic on Palestinian healthcare professionals, including their emotions, perceived stressors, and coping strategies, is lacking and has been disregarded during the pandemic. Palestinian health workers directly involved in the diagnosis and treatment of patients with COVID-19 in health centers and hospitals are at risk of developing psychological and mental health issues. The challenges of accessing adequate protective equipment, the burden of work, the lack of treatments available, the risk of contracting an infection and having to stay in quarantine, the lack of experience and training in communicable disease management, and the rising number of cases and fatalities all increase the risk of mental health issues that may have an impact on the psychological well-being of health staff [10].

Stress is defined as a situation that an individual perceives to be personally significant, with demands that exceed the individual’s coping resources [20]. If environmental demands are deemed hazardous, an individual evaluates their available coping resources to determine whether they believe they are capable of dealing with the stressor (i.e., a stressor). Coping refers to the thoughts and behaviors that people employ in order to deal with the internal and external demands of a stressful event [20]. Having effective coping strategies during pandemic outbreaks is critical for protecting healthcare professionals from infection as well as their involvement in preventing many stress-related psychiatric problems [17,21]. During a pandemic outbreak at the workplace, it is also important to assess the mental health status and needs of healthcare workers.

The act of coping with stress motivates individuals to engage in certain coping behaviors [15]. A person’s motivation is the factor that leads to their satisfaction or dissatisfaction prior to making a decision, according to Herzberg (1968) [16]. Therefore, it may influence coping strategies by regulating the appraisal or experience of stress. Effective coping strategies, as well as social and emotional support, undoubtedly influence the motivation of healthcare personnel during pandemic outbreaks [22]. During the first wave of the COVID-19 pandemic, HCWs reported high levels of stress and emotional trauma which affected their motivation and coping strategies [17]. As a result, health organizations use corporate incentives, awards, and recognition as motivational strategies to motivate HCWs. This category includes all financial and nonfinancial incentives, awards, and recognition used to motivate employees [23]. Furthermore, during extremely difficult times, positive and negative emotions coexist [24,25,26]. Positive emotions support adaptive stress adjustment and recovery from stressful situations, according to research on stress encounters and coping [24,26,27,28,29]. Frontline medical staff members in pandemics experience increased emotional turmoil as a result of caring for COVID-19-infected patients [30,31] (Cai et al., 2020; Khalid et al., 2016). Furthermore, healthcare workers do not always have the skills needed to deal with such acute stressors, which frequently leads to disengagement, avoidance, and emotional suppression [31]. A study suggested that healthcare professionals working in a COVID-19 ward should receive psychological support in the form of “timely psychological assistance”, as well as training in coping strategies. Researchers concluded that nurses might benefit from training in regulating their emotions and developing a better coping strategy in situations that demand resilience to combat the pandemic [18].

In Palestine, the first cases of COVID-19 were reported in early March 2020 in the Bethlehem Governorate and the outbreak quickly overwhelmed the health services. Despite the nationwide lockdown imposed on the 5th of March, the number of cases and deaths accelerated. By January 2021, the Ministry of Health had recorded 183,365 cases and 2072 deaths in the country [19].

The complicated political, social, and economic settings prior to the COVID-19 outbreak, as well as the pandemic and quarantine, had a significant impact on the mental health outcomes of HCWs [32]. For example, given the fragile healthcare system and complicated political circumstances, the COVID-19 outbreak added additional challenges and difficulties for Palestinian healthcare workers. Furthermore, the COVID-19 crisis exposed significant gaps in Palestine’s social and public health systems, including social exclusion, inequalities, fragility, unpreparedness, underinvestment, and a severe shortage of COVID-19 tests, sanitation, hygiene supplies, ventilators, and ICU beds [33]. Working in a healthcare system that was severely constrained by Israeli military occupation showed an increased risk of stress and burnout among healthcare professionals. Several studies in Palestine looked into HCWs’ stress, fear, and anxiety. During the COVID-19 pandemic, HCWs reported a high level of fear and stress, according to El Sharif et al [34]. Half of the HCWs were afraid when caring for COVID-19 patients; half were afraid of becoming infected while caring for COVID-19 patients; and 90% were afraid of passing the infection on to their families. Another study (2020) found that 74.0% of Palestinian HCWs experienced high levels of stress during the outbreak. The main source of stress (91.6%) was the fear of infecting family members [35].

However, evidence about the impact of the COVID-19 pandemic on Palestinian healthcare professionals, including their emotions, perceived stressors, and coping strategies, is lacking and has been disregarded during the pandemic. Palestinian healthcare workers, directly involved in the diagnosis and treatment of patients with COVID-19 in health centers and hospitals, are at risk of developing psychological and mental health issues. The challenges of accessing adequate protective equipment, the burden of work, the lack of treatments available, the risk of contracting an infection and having to stay in quarantine, the lack of experience and training in communicable disease management, and the rising number of cases and fatalities all increase the risk of mental health issues that may have an impact on the psychological well-being of health staff [10].

In order to create interventions that can lessen psychological stress during the pandemic outbreak at the workplace, it is important to assess the mental health status and needs of healthcare workers to develop interventions that can reduce psychological stress. Therefore, the aims of this study are to describe the HCWs’ emotions, factors causing stress, and strategies used to reduce stress and to examine the relationships between HCWs’ level of stress and their coping and motivational strategies during the COVID-19 pandemic in Palestine.

## 2. Materials and Methods

### 2.1. Procedure

This study was conducted among healthcare staff working in healthcare facilities in the West Bank, health quarantine centers in the Gaza Strip, and East Jerusalem hospitals dealing with COVID-19 patients between October 2020 to February 2021.

A cross-sectional study was conducted in the major healthcare facilities, which included the COVID-19 hospitals and quarantine centers. The participants were selected from 12 COVID-19 healthcare facilities: eight hospitals and four COVID-19 centers in the West Bank, one COVID-19 hospital in the Gaza Strip, and three hospitals in East Jerusalem. An anonymous online self-administered survey method was used for data collection. An electronic version of the questionnaire using Google forms with an introductory invitation was sent to the targeted participants. Due to closure and movement restrictions, field coordinators obtained participants’ WhatsApp numbers from personnel departments and sent the questionnaire web link to them.

### 2.2. Subjects

This study targeted doctors and nurses who work directly with patients in COVID-19 hospitals and quarantine centers in Palestine. The total number of HCWs (700 HCWs) was obtained from the targeted healthcare facilities. Specialized, residents, general physicians, nurses, and nursing assistants were invited to participate in this survey. Of them, 506 completed the questionnaire. Therefore, the study response rate was 72.3%.

### 2.3. Tool and Measures

In this study, participants were asked to answer a self-reported questionnaire based on the one developed previously by Lee et al. (2005) [36], modified by Khalid et al. (2016) [30], which was previously used for Middle East Respiratory Syndrome (MERS) due to the lack of a generic questionnaire to be used in outbreaks, pandemics, and emergency situations like COVID-19. The questionnaire was translated into Arabic and then translated back to English. Its terminology was checked by an expert in mental health and was then piloted by 20 healthcare workers to determine the accuracy and clarity of the terminology used in the Arabic language. It consisted of five sections with 72 questions (i.e., emotions, factors causing stress, factors helping to reduce stress, coping strategies, and motivational strategies). Cronbach’s α was calculated for each section to ensure the internal consistency of the questionnaire.

A socio-demographic data sheet was included to collect information related to the participant’s age, gender, education, setting, specialty, income, place of residence, and place of work. Information on previous experience of COVID-19, previous courses or experience of communicable diseases, training in the management of COVID-19 patients, and duration of training for COVID-19 patients’ care and treatment were also collected.

**Figure 1:**
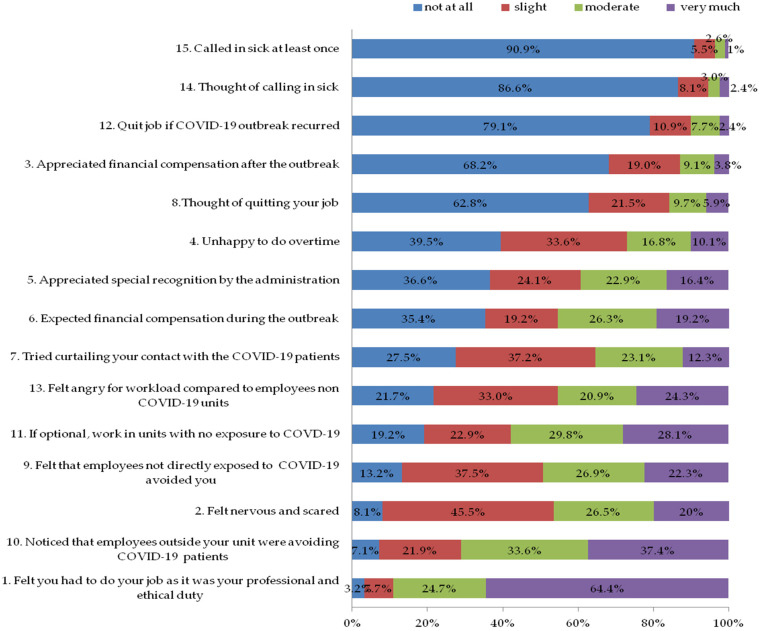
Staff emotions during COVID-19 outbreak.

The first section of the questionnaire explored the emotion of staff during the COVID-19 outbreak and it consisted of 15 questions (questions 1 to 15, Figure 1). Each question had a 4-point scale (0 = not at all; 1 = slight; 2 = moderate; 3 = very much). The internal consistency coefficient was 0.69 (Cronbach’s α) for the severity of feelings.

**Figure 2:**
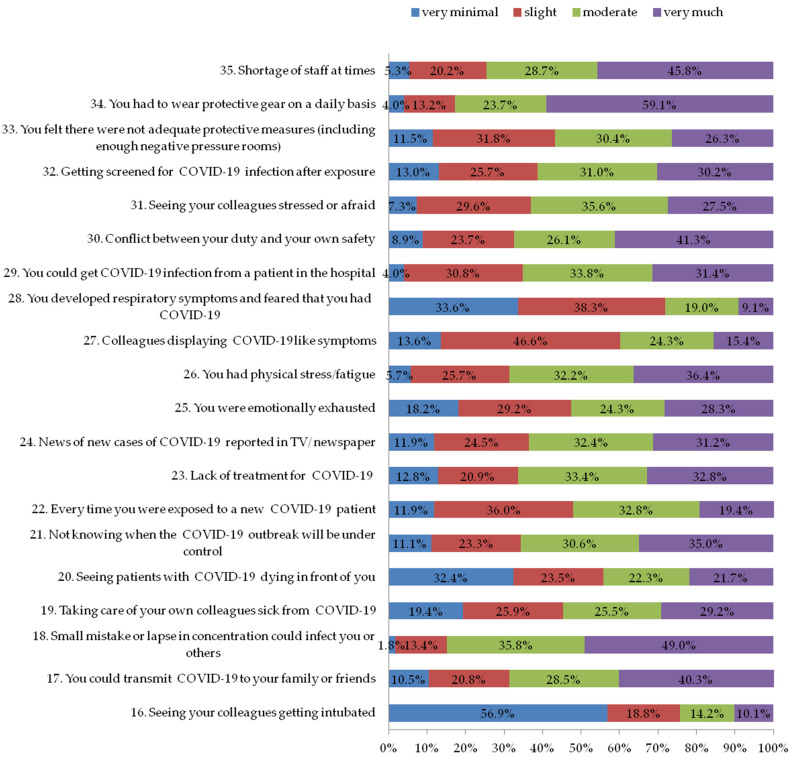
Factors that caused stress among staff during COVID-19 outbreak.

The second section of the questionnaire explored factors that could have caused stress among staff and it consisted of 20 questions (questions 16 to 35, Figure 2). Each question had a 4-point scale (0 = very minimal; 1= slight; 2 = moderate; 3 = very much). The internal consistency coefficient was 0.89 (Cronbach’s α) for the stress severity.

**Figure 3:**
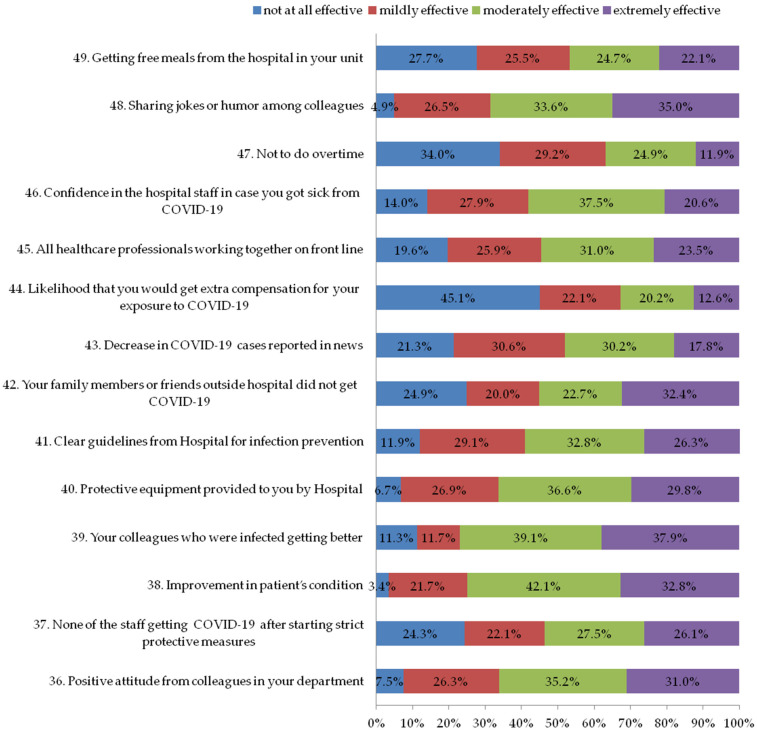
Factors that helped to reduce stress during COVID-19 outbreak.

The third section of the questionnaire explores the factors available to hospital staff, either directly or indirectly, that could have helped to reduce their stress and it consisted of 14 questions (questions 36 to 49, Figure 3). Each question had a 4-point scale (0 = not at all effective; 1 = mildly effective; 2 = moderately effective; 3 = extremely effective). The internal consistency coefficient (Cronbach’s α) was 0.80 for the degree of effectiveness.

**Figure 4:**
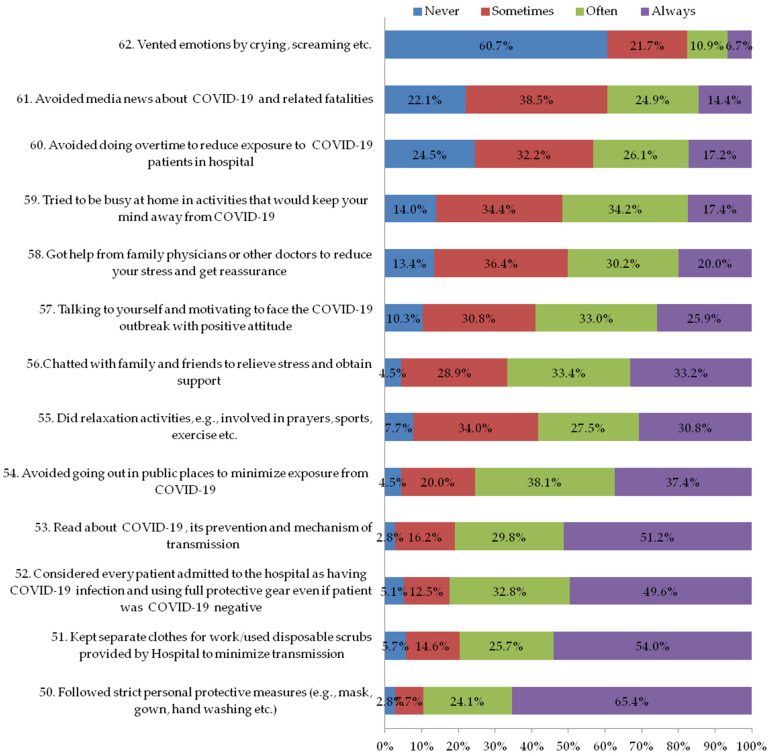
Personal coping strategies staff could have used during COVID-19 outbreak.

The fourth section of the questionnaire explored the personal coping strategies that staff could have used and it consisted of 13 questions (questions 50 to 62, 72, Figure 4). Each question had a 4-point scale (0 = never; 1 = sometimes; 2 = often; 3 = always). The internal consistency coefficient was 0.83 (Cronbach’s α) for the rating of coping strategies.

**Figure 5:**
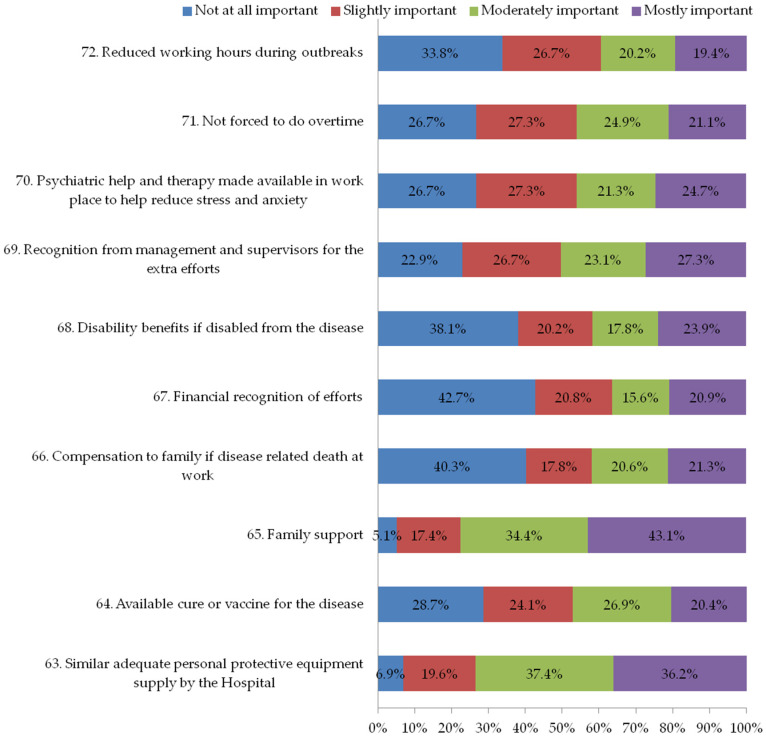
Motivational factors to encourage the continuation of work in future outbreaks.

The fifth section of the questionnaire explored the motivational factors to encourage the continuation of work in future outbreaks and it consisted of 10 questions (questions 63 to 72, Figure 5). Each question had a 4-point scale (0 = not at all important to 4 = most important). The internal consistency coefficient was 0.76 (Cronbach’s α) for the rating of motivation strategies.

The questionnaire was piloted on 15 respondents to determine its comprehensibility, face validity, and estimated completion time, which led to some refining of a few questions in the socio-demographic data sheet to enhance the questionnaire.

### 2.4. Ethical Approval and Consent to Participate

All methods in this study were performed in accordance with the Declaration of Helsinki. The study was approved by the Palestinian Ministry of Health Ethical Committee (REF: HEUGD20190) and Al Quds University Research Ethical Committee (Ref No. 130/Rec/2020). This online survey was anonymous. Written information about the purpose of the survey and how the data would be used was provided at the beginning of the survey. Upon filling out the questionnaire, individuals provided informed consent for participation in this study.

### 2.5. Data Analysis

SPSS version 25 (IBM Corp., Chicago, IL, USA) was used for the data analysis. Frequencies, means, and standard deviation analyses were used as needed. Cronbach’s α was also performed to evaluate the internal consistency of the questionnaire’s five sections.

To assess HCWs’ stress level, the survey section two contained 20 questions with a four-point scale which were combined into one score; i.e., the stress score. This index was very important in our study because it enabled us to determine the association between the stress index and the coping and motivation strategies. We calculated means, medians, standard deviations (SD), and interquartile ranges for each score. An inferential analysis was performed based on the interquartile ranges of a stress scale to define a stress index: low stress (score ≤ 27), moderate stress (score 28–43), and high stress (score > 43).

To assess coping strategies, the survey tool included 13 questions with a four-point scale that were combined into one score; i.e., the coping strategies score. Similarly, a motivation score was developed from the 10 questions with a four-point scale that were combined into one score. We calculated means, medians, and standard deviations (SD) for each score. Spearman’s rho was used to analyze the correlation between stress scoring and coping strategies scoring, and the correlation between stress scoring and motivation scoring. The *p*-value cutoff for Spearman’s rho was 0.05.

After adjusting for demographic and experience factors, two models for coping strategies and motivational factors were developed with the stress scoring index, and results were presented as adjusted OR (AOR) with 95% CI. Additionally, a separate multivariate model was developed for the coping and motivation factors scorings with the stress scoring index after controlling for the same demographic and experience variables.

## 3. Results

### 3.1. Demographic Characteristics of the Participants

In total, 506 participants filled in the study’s online survey. More than half of the participants (n = 276, 54.5%) were younger than 30 years old, married (n = 309, 61.1%), and mostly males (n = 429, 84.8%). The survey was completed by 158 (31.1%) doctors and 347 (68.7%) nurses. Of the sample, 60% (n = 303) were from the West Bank, 28.5% (n = 144) were from Gaza Strip, and 11.7% (n = 59) were from East Jerusalem. Most participants (n = 426, 84%) had a monthly salary of less than 1500 US dollars per month and lived mostly in cities and refugee camps (Table 1).

According to Table 1, 61.1% (n = 309) of participants worked in COVID-19 special treatment centers. Additionally, they reported that their work there was compulsory (n = 370, 73%). Most of them were working with these patients for the first time (76.9%), but only half of them were trained on how to deal with COVID-19 patients.

### 3.2. Section One: Staff’s Emotion

The survey asked about staff emotions during the COVID-19 outbreak (Figure 1). According to the findings, most of the participants felt nervous and scared to perform their job (slightly, moderately, and very much combined) (92%), they felt they had an ethical and professional duty to continue working during the pandemic (96.8%), employees outside their units avoided COVID-19 patients (92.9%), and 86.7% thought that employees not directly exposed to COVID-19 avoided them.

### 3.3. Section Two: Factors That Caused Stress among Staff during the COVID-19 Outbreak

The second section of the questionnaire looked at stress factors, staff safety, and the safety of colleagues and family members (Figure 2). Results showed that most participants answered “slightly, moderately and very much” to most questions. They were worried about their own safety: 98.2% of them said that a minor mistake or lapse in attention could lead to them becoming infected or spreading it to others; and 96.0% said that they feared they might become infected with COVID-19 from a hospital patient.

The stress score had a median and quartile of 35 (27–43), and a mean and standard deviation (±SD) of 35.1 ± 11.2. The average stress score was 49% of the test’s highest possible score. About 24.1% of the participants had a high stress score of 43 or higher (upper quartile) and 25.7% had a score of 27 or lower (lower quartile).

### 3.4. Section Three: Factors That Reduce Stress among Staff during the COVID-19 Outbreak

For the factors that directly or indirectly help to reduce stress during the COVID-19 outbreak, Figure 3 indicates that 96.6% of the participants (mildly, moderately, and extremely) reported that a patient’s improvement reduced stress, as well as sharing jokes or humor with colleagues (95.1%), using protective equipment provided by the hospital (93.3%), and having a positive attitude from their colleagues in their department (92.5%).

### 3.5. Section Four: Personal Coping Strategies Staff Could Have Used during the COVID-19 Outbreak

Most of the participants used coping strategies that prevented them from contracting the disease (Figure 4). For example, 97.2% of the participants followed strict personal protective measures (e.g., mask, gown, hand washing, etc.); read about COVID-19, its prevention, and forms of transmission (97.2%); avoided going out in public places to minimize exposure to COVID-19 (95.5%); and avoided media news about COVID-19 and related fatalities (77.9%).

### 3.6. Section Five: Motivational Factors to Encourage the Continuation of Work in Future Outbreaks

The fifth section asked participants about motivators to continue working during future infectious disease outbreaks. Family support (94.9%) was the most important motivator, followed by adequate personal protective equipment supplied by the hospital (93.1%) and recognition from management and supervisors for the extra efforts of staff (77.1%).

### 3.7. Associations between Respondent Characteristics, Experience with COVID-19, Coping Strategies with Stress Score Index

Comparing the various stress scoring index (low, medium, high) with demographic and experience variables (Table 2), younger HCWs had significantly increased stress levels (AOR: 1.56, 95%CI: 1.03–2.35, *p* < 0.05) compared to older adults, with physicians reporting less stress than nurses (AOR: 0.76, 95%CI: 0.67–0.97) *p* < 0.05). In addition, receiving no training on the treatment of COVID-19 was strongly associated with stress (AOR: 1.36, 95%CI: 1.20–1.54, *p* < 0.05). Other factors, however, did not show any significant association with the stress index (Table 2).

### 3.8. Association between Coping Strategies, Motivation with Stress Scoring Index

In comparing stress scoring index (low, medium, high) and coping strategies, HCWs considered every patient admitted to be infected with the COVID-19 infection and used full protective gear even if the patient was negative for COVID-19. The stress scoring index was significantly associated with avoiding public places to minimize COVID-19 exposure, avoiding news about COVID-19 and related fatalities, and crying and screaming to vent emotions (*p* < 0.05) (Table 3).

Furthermore, the availability of a possible cure for the disease, family support, recognition from management and supervisors for the extra effort put in during the pandemic, compensation to the family if disease-related death occurred at work, financial recognition of efforts, and reducing working hours during outbreaks were significantly inversely associated to stress score (Table 3).

### 3.9. Correlation between Stress Scoring, Coping, and Motivation Factors Scoring

A significant positive correlation was found between the stress and the coping scores (R = 0.25), but not with the motivation score. A correlation of 0.16 was found between coping and motivation factors (Spearman’s rho, *p*-value < 0.05). Furthermore, only coping strategies were significantly associated with stress index (Table 4).

## 4. Discussion

This study is the first to examine the emotions, perceived stressors, and coping and motivation factors of Palestinian healthcare workers who faced the deadly COVID-19 outbreak in Palestine. Palestinian HCWs (doctors and nurses) experienced emotional turmoil during the COVID-19 pandemic, particularly a high level of stress, with an emphasis more on personal and social safety. Approximately 24.1% had a high level of stress, and 25.7% had a low level of stress. These results were similar to those of other studies that revealed high levels of stress [36,37,38,39]. Other studies reported a low prevalence of stress (e.g., 21.9%) [40,41,42].

According to the study, Palestinian healthcare workers experienced emotional turmoil during the COVID-19 epidemic. During the epidemic, the main emotional factor was the ethical and professional obligation to continue working. Similarly, Khalid et al. found that healthcare workers’ professional and ethical obligations drove them to continue working [30]. Healthcare workers expressed concern about prioritizing resources and complying with patient needs, especially when resources are limited and staff have to deny services to a patient [43]. By working during an epidemic, healthcare workers may feel powerless and under extreme pressure to perform their professional and ethical duties [44]. Other emotions occurred because employees outside their units avoided COVID-19 patients, and healthcare workers thought that employees not directly exposed to COVID-19 avoided them. A study conducted by Khalid et al. (2016) revealed that even though employees expected extra compensation and special recognition during a disaster, they did not report that these incentives were the most significant motivators for continuing to work [30]. Palestinian healthcare workers may have been avoiding others due to the fact that COVID-19 is highly contagious and can be spread easily through respiratory droplets and interpersonal contact [1].

Age, occupation, and getting training are the only three variables that are associated with stress when examining the relationship between stress and respondents’ characteristics and experiences in this study. For example, the findings of this study indicated that younger HCWs are more likely to experience stress than older workers due to their age. Study results from Saudi Arabia [42] and Indonesia [41] show that younger workers experience significantly more stress than older workers. Participants who were older and had more years of work experience, on the other hand, had more stable mental health, were less distracted under stress and were relatively free of neurotic anxiety [41,45]. Therefore, it is very likely that young Palestinian HCWs may feel stressed and anxious since they are not familiar with infectious disease outbreaks such as the COVID-19 pandemic. In our study, physicians experienced less stress than nurses. A study in Polanda found that nurses were more stressed at work during the COVID-19 pandemic [46] because nurses provided direct patient care while doctors and other professionals spent less time with patients. However, Suratman et al. (2020) [47] found that physicians felt the most stress compared to nurses and midwives (33.3%).

Furthermore, there was a significant association between having trained for treating COVID-19 patients and having higher levels of stress than those who did not receive such training. Elbqry et al. (2021), found a significant relationship between COVID-19 psychological stress levels and HCWs satisfactory level of knowledge [47]. HCWs who were confident in infection control reported the lowest level of stress [48]. According to Elbqry et al. (2021), HCWs awareness of infection prevention and control measures, effective communication, and proper information dissemination would have an impact on reducing stress and fear [47]. The main factors creating stress among the participants in the current study were their own safety, COVID-19 infection, and the possibility of infecting others. These undesirable findings could be attributable to a lack of basic personal protective resources. Fear of a shortage of personal protective equipment is widespread among healthcare workers worldwide [49,50] particularly in impoverished countries such as Palestine that lack a robust healthcare infrastructure [51]. During the COVID-19 pandemic in Palestine, a study by Alser et al. (2021), reported a lack of protective equipment such as alcohol-based hand sanitizers, gloves, face masks, eye protection, isolation gowns, and face shields, as well as a lack of adequate training on local protocols of COVID-19 in health care settings [52]. Therefore, having basic personal protective resources may reduce stress and panic [1]. Furthermore, healthcare workers must prioritize not transmitting the infection to vulnerable groups such as family members, the elderly, people with weakened immune systems, and young children [51,52,53,54,55]. As a result, as a precautionary measure, healthcare workers limited their exposure to the general public and to their family members [51,55].

The development of interventions for this high-risk population to reduce the effects of the COVID-19 outbreak on mental health depended on understanding the stress-reduction techniques utilized by HCWs. In the current study, among the stress-reduction strategies, were treating all patients admitted to the hospital as having COVID-19 infection and wearing full protective gear, avoiding public places to limit COVID-19 exposure, and venting emotions through crying and screaming. These findings are similar to those of the Khalid et al. (2016), study and reflect professional healthcare workers’ extreme caution in dealing with COVID-19 as a new disease with devastating global implications [30]. However, Windarwati et al. (2021), found that adopting a positive attitude to motivate themselves (98.3%), reading about COVID-19 and its prevention and transmission (98.3%), and using appropriate self-protection measures (mask, gown) (98.3%) were the three most common coping strategies used by health workers to deal with the COVID-19 pandemic [49].

Interestingly, HCWs in the current study vented their emotions by crying and screaming as a coping strategy. According to studies, the majority of physicians (57% and 53.1%) have cried at work [56,57]. Other studies have found that nurses are more likely than physicians to agree with the statement “I vented emotions by crying, screaming etc.” as a coping strategy [17]. Crying has been shown to promote emotional healing for stress management, improve mood by stimulating endorphin secretion, and reduce pain [58]. Other studies reported negative attitudes towards crying in the workplace, particularly in the presence of a patient [59,60], because it does not benefit the patient, and may increase the physician’s risk of stress and burnout [61]. According to the Veronese et al. study, (2022), a positive correlation has been found between the stress of COVID-19 and burnout and trauma among Palestinian HCWs, saying that they have daily trauma experiences due to Israeli military violence [62]. Consequently, workplace supervision and advising and supporting HCWs in dealing with their emotions may be an option for overcoming feelings of uncertainty and inability [63]. As a coping strategy, Vicente et al. (2021), suggest defusing conversations with other health professionals when in difficult situations in order to release emotions. Furthermore, employers must implement preventative measures, such as training programs, in the workplace before traumatic events such as COVID-19 outbreaks or future pandemics to control HCWs’ negative emotions [64]. 

Furthermore, one of the objectives of the current study was to identify the motivational factors used by Palestinian HCWs in the face of the COVID-19 pandemic. The available cure or vaccine for the disease, family support, compensation to the family if disease-related death at work, financial recognition of efforts, recognition by management and supervisors of extra efforts, and reduced working hours during outbreaks were the main factors that kept the participants motivated during the pandemic. Cai et al. (2020), found in their study that medical staff expected to be recognized by hospital authorities [31]. However, Khalid et al. (2016) showed that extra pay, special recognition, and avoidance of overtime during a disaster were not reported as the most important incentives to continue work [30]. Other studies, however, showed that safety, understanding of the disease, special compensation, and recognition were the major motivators [4,30,63,64,65]. Surprisingly, the availability of a cure or vaccine for the disease was a motivator related to stress reduction. However, Kabamba, (2020), found that only 27.7% of healthcare workers would accept a COVID-19 vaccine if it became available [66], whereas Shekhar et al. (2021) found that 36% of healthcare workers in the United States of America were willing to take the vaccine as soon as it became available [67]. In China, Cai et al. (2020) found that health professionals were unconcerned about the lack of COVID-19 treatment [31]. In addition, the current study’s findings indicated the significance of family support in dealing with infected patients’ psychological problems which are similar to the findings of the Windarwati et al. (2021), study [49]. This finding may highlight the importance of providing formal mental health services to Palestinian healthcare staff in the workplace, including hospitals, during a pandemic and other future pandemics rather than relying on staff’s families. Liu et al. (2021), emphasized the importance of mental health professionals collaborating with healthcare workers in intensive units to reduce stress and the risk of depression. Seeking assistance from family and friends was an important supportive measure [63]. Cai et al. (2020), reported that HCWs were uninterested in reducing stress by consulting a psychologist or other doctors and medical technicians to discuss their emotions [31]; therefore, to address the mental health needs of employees, various approaches were developed, including online materials, a psychological assistance hotline, and group activities for stress reduction. A rest area was set up to provide food, recreational activities, and regular visits to the rest area by a counselor [49,68]. Kang et al. (2020), found that telephone helplines can help reduce mental problems among healthcare workers [69].

Moreover, previous research [30,31,70] investigated the relationship between stress and coping strategies. Reduced stress levels were found to be associated with the use of coping strategies [71,72]. The current study found a weak significant relationship (r = 0.25) between stress and coping strategies. Previous research found no link between coping strategies and stress [57], whereas Ali et al. (2021), found a strong negative correlation between coping strategies and stress [22]. One possible explanation for our study’s weak relationship between stress and coping strategies is that the majority of participants used avoidance as a coping strategy, which may have increased their stress. In the study by Babore et al. (2020), stress increased as the use of avoidance strategies increased [73]. Another possible explanation is that Palestinian healthcare professionals may not receive adequate training and support to deal with stress and pressures in the workplace, which would have a negative impact [74]. According to one study, the most common strategies used by HCWs when faced with extremely stressful situations were maintaining a normal life, considering solutions, maintaining situational control, and searching for information [75]. Another study discovered that the two most common coping strategies used by healthcare professionals were acceptance of the dire circumstances and maintaining a cheerful outlook while working [76]. As a result, policymakers and managers must conduct training and provide interventions on how to manage mental health issues and cope with stress in various situations, including pandemics, in order to assist HCWs in developing diverse adaptive coping strategies and avoiding the use of unhealthy coping strategies.

Finally, employee motivation is critical for organizations to achieve any set of objectives [77]. Employee motivation boosts productivity, which helps businesses survive and thrive [78]. The current study’s findings did not reveal a significant relationship between stress and motivation factors. In contrast, Park et al. (2012), found that stress may be related to motivation, whereas Khalatbaria et al. found a positive relationship between stress and job motivation [79]. Chen et al. (2012), discovered a significant relationship between motivation and work stress in their study of Taiwanese workers. They were unable to find a link between work motivation and stress among workers in Mainland China. There is evidence that employees who are under stress are less motivated, which could explain why the current study found no meaningful correlation between stress and motivation factors [80]. The study by Saleem et al. (2015), shows that perceived stress has a negative impact on employees’ motivation [81], and the study by Chew et al. (2013), confirms this viewpoint [82]. Chen et al. (2012), advised managers to conduct work motivation scale tests when hiring and selecting employees in order to avoid hiring people who are unmotivated because they are more likely to experience high levels of stress at work when they first start working for the organization [80]. Furthermore, effective stress management may promote motivation, and efforts to increase motivation may benefit significantly from stress interventions [79]. Furthermore, management and administration are responsible for creating a relaxing and stress-free working environment for their employees so that they can continue to perform at their best [15].

## 5. Study Limitations

Our study had some limitations. The survey took place during the second peak of the pandemic and under a strict lockdown. In this period, HCWs experienced extreme stress at work and on a personal level, which may exaggerate their responses. Another limitation of this study is that obtaining data was done through self-report questionnaires which makes it liable for reporting bias. Furthermore, convenience sampling and cross-sectional design are barriers to making casual conclusions. Nevertheless, despite these limitations, providing our findings about the consequences of the COVID-19 pandemic on the mental health of HCWs in Palestine represents a valuable contribution to the literature

## 6. Implications of the Study

Health workers are at the forefront of dealing with health problems due to the COVID-19 outbreak. However, using PPE everyday causes discomfort, so it becomes a stressor for health workers to be provided adequate personal protective equipment (PPE) training to increase safety and comfort, being provided adequate support to increase their productivity and keep them motivated are also among strategies to reduce stress among HCWs. Family support is also one of the key factors that motivate healthcare workers to provide healthcare services during the COVID-19 outbreak. Therefore, it is important to keep contact between HCWs and their families, in addition to proving psychological support and interventions to decrease their stress during the pandemic. The availability of adequate information, recognition, financial compensation, and support from hospitals also helped motivate the health workers to deal with the outbreak.

One of utmost importance for healthcare institutions, policymakers, and managers is to prepare for the possibility of another epidemic/pandemic. Therefore, improvement of the clinical environment, such as the availability of protective equipment, training, providing information, and focusing on those with a high risk for exposure such as young HCWs or high-risk settings, are important to face future challenges of pandemics similar to COVID-19.

Future research is needed to investigate the effectiveness of different psychological interventions that improve motivation and the use of coping strategies during stressful pandemics among HCWs.

## 7. Conclusions

The emotions of Palestinian healthcare workers, their perceived stressors, and coping strategies showed some unique elements. The results of the study show that Palestinian healthcare workers experienced emotional turmoil during the COVID-19 pandemic at the workplace, particularly young adult HCWs, nurses, and those who did not receive training on the treatment of COVID-19. Avoidance was the participant’s primary coping mechanism, and their concern for their own safety and having the COVID-19 infection was also a significant source of stress. These results indicate the need to provide supervision and psychological counseling and intervention to professional healthcare workers who work directly with COVID-19 patients in health settings during the current pandemic or in a future outbreak to increase resilience and reduce the dependence on families and others for psychological support. Policymakers and managers must also conduct training and provide interventions on how to cope with stress in pandemics, in order to assist HCWs in developing their adaptive coping strategies and increase their motivation. Finally, basic personal protective resources equipment such as alcohol-based hand sanitizers, gloves, face masks, isolation gowns, and face shields should be available in any future pandemic to reduce stress and panic among HCWs.

## Figures and Tables

**Table 1:** Participants’ demographic characteristics and experience with COVID-19.

		F	%
Gender	Female	77	15.2%
	Male	429	84.8%
Age	20–30 years	276	54.5%
	31–40 years	158	31.2%
	41–50 years	60	11.9%
	>50 years	12	2.4%
Marital Status	Single	190	37.5%
	Married	309	61.1%
	Divorced	7	1.4%
Place of Residence	Village	212	41.9
	City	232	45.8
	Camp	62	12.3
Occupation	Physician	158	31.3%
	Nurse	348	68.7%
Area	West Bank	303	59.9%
	Jerusalem	59	11.7%
	Gaza Strip	144	28.5%
Monthly Income	<1000$	164	32.4%
	1000–1500$	262	51.8%
	1500–2100$	67	13.2%
	>2100 $	13	2.6%
Place of COVID-19 work	Special treatment centers	139	27.5%
	Hospitals	309	61.1%
	Quarantine hotels	8	1.6%
	Work in more than one treatment place	50	9.9%
Was this your first time working with COVID-19 patients?	No	117	23.1%
	Yes	389	76.9%
Did you receive training to deal with COVID-19 patients?	No	310	61.3
	Yes	196	38.7
Did you receive training to treat COVID-19 patients?	No	286	56.5%
	Yes	196	38.7%
	Can not remember	24	4.7%
Your work with COVID-19 patients in treatment centers is	Compulsory	370	73.1%
	Voluntary	96	19.0%
	Other	40	7.9%

**Table 2:** Associations between respondents’ characteristics, experiences, and stress scoring index during COVID-19 pandemic.

		Stress Index							Adjusted Analysis *			
		≤27 Low n= 130		28–43 Moderate n = 254		>43 High n = 122		*p* Value			95% Wald Confidence Interval for Exp (B)	
		n	%	n	%	n	%	Sig.	Sig.	Exp (B)	Lower	Upper
Age	20–30 years	50	38.5	149	58.7	77	63.1	**0.02**	0.034	0.25	0.07	0.90
	31–40 years	54	41.5	72	28.3	32	26.2		0.073	0.54	0.27	1.05
	41–50 years	20	15.4	29	11.4	11	9.0		0.002	0.46	0.29	0.75
	>50 years	6	4.6	4	1.6	2	1.6			Ref.		
Occupation ††	Physician	55	42.3	76	30.0	27	22.1	**0.007**	0.00	2.38	1.14	4.97
	Nurse	65	50.0	160	63.2	81	66.4		0.01	2.14	1.34	3.41
	Other	10	7.7	17	6.7	14	11.5			Ref.		
Place of COVID-19 work	COVID-19 treatment centers	45	34.6	72	28.3	22	18.0		0.19	1.66	0.86	3.20
	COVID-19 hospitals	67	51.5	154	60.6	88	72.1	0.057	0.81	0.84	0.21	3.26
	COVID-19 quarantine hotels	3	2.3	4	1.6	1	0.8		0.03	1.58	1.03	2.43
	More than one treatment place	15	11.5	24	9.4	11	9.0			Ref.		
Did you receive training to treat COVID-19 patients?	No	53	40.8	148	58.3	85	69.7	**000**	0.74	0.84	0.30	2.35
	Yes	69	53.1	99	39.0	28	23.0		0.001	0.41	0.25	0.68
	Can’t remember	8	6.2	7	2.8	9	7.4			Ref.		

* Multivariate Model included gender, age, marital status, place of residence, occupation, †† place of work, income level, first time working with COVID-19 patient, receive training to deal with COVID-19 patients, receive training to treat COVID-19 patients, and work with COVID-19 patients in treatment centers. Significant *p* values are in bold.

**Table 3:** Associations between respondent coping strategies and motivation factors with COVID-19 and stress scoring index during COVID-19 pandemic.

		Stress Index							Adjusted Analysis *			
		≤27 Low n = 130		28–43 Moderate n = 254		>43 High n = 122		*p* Value			95% Wald Confidence Interval for AOR	
		n	%	n	%	n	%	Sig.	Sig.	AOR	Lower	Upper
Model 1: Coping strategies *												
52. Considered every patient admitted to the hospital as having COVID-19 infection and using full protective gear even if patient was COVID-19 negative	Never	8	6.2	10	3.9	8	6.6	**0.09**	**0.91**	**0.99**	**0.72**	**1.35**
	Sometimes	21	16.2	32	12.6	10	8.2		**0.04**	**0.79**	**0.63**	**0.99**
	Often	51	39.2	85	33.5	30	24.6		**0.01**	**0.84**	**0.72**	**0.97**
	Always	50	38.5	127	50.0	74	60.7			Ref.		
54. Avoided going out in public places to minimize exposure from COVID-19	Never	13	10.0	7	2.8	3	2.5	**0.002**	**0.04**	**0.67**	**0.48**	**0.94**
	Sometimes	34	26.2	49	19.3	18	14.8		0.29	0.87	0.71	1.07
	Often	47	36.2	99	39.0	47	38.5		0.92	0.99	0.85	1.17
	Always	36	27.7	99	39.0	54	44.3			Ref.		
61. Avoided media news about COVID-19 and related fatalities	Never	33	25.4	56	22.0	23	18.9	**0.000**	0.50	0.93	0.74	1.18
	Sometimes	65	50.0	94	37.0	36	29.5		0.01	0.80	0.65	0.99
	Often	19	14.6	72	28.3	35	28.7		0.57	0.94	0.75	1.16
	Always	13	10.0	32	12.6	28	23.0			Ref.		
62. Vented emotions by crying, screaming etc.	Never	87	66.9	157	61.8	63	51.6	**0.002**	0.03	0.76	0.59	0.99
	Sometimes	29	22.3	56	22.0	25	20.5		0.33	0.87	0.66	1.15
	Often	8	6.2	30	11.8	17	13.9		0.93	0.99	0.73	1.33
	Always	6	4.6	11	4.3	17	13.9			Ref.		
Model 2: Motivation **												
64. Available cure or vaccine for the disease	Never	24	18.5	74	29.1	47	38.5	000	0.07	1.22	0.981	1.51
	Sometimes	41	31.5	62	24.4	19	15.6		0.80	0.97	0.77	1.21
	Often	48	36.9	65	25.6	23	18.9		0.19	0.86	0.70	1.07
	Always	17	13.1	53	20.9	33	27.0			Ref.		
65. Family support	Never	8	6.2	9	3.5	9	7.4	0.001	0.73	0.95	0.71	1.26
	Sometimes	28	21.5	49	19.3	11	9.0		0.003	0.76	0.63	0.91
	Often	55	42.3	85	33.5	34	27.9		0.05	0.86	0.74	1.00
	Always	39	30.0	111	43.7	68	55.7			Ref.		
66. Compensation to family if disease-related death at work	Never	52	40.0	101	39.8	51	41.8	0.000	0.22	0.85	0.66	1.08
	Sometimes	29	22.3	49	19.3	12	9.8		0.34	0.88	0.68	1.19
	Often	29	22.3	58	22.8	17	13.9		0.42	0.90	0.71	1.15
	Always	20	15.4	46	18.1	42	34.4			Ref.		
67. Financial recognition of efforts	Never	49	37.7	117	46.1	50	41.0	000	0.020	0.75	0.58	0.97
	Sometimes	42	32.3	52	20.5	11	9.0		0.004	0.70	0.55	0.89
	Often	24	18.5	38	15.0	17	13.9		0.080	0.81	0.64	1.03
	Always	15	11.5	47	18.5	44	36.1			Ref.		
69. Recognition from management and supervisors for the extra efforts	Never	22	16.9	57	22.4	37	30.3	**0.023**	0.01	1.40	1.07	1.81
	Sometimes	39	30.0	65	25.6	31	25.4		0.03	1.26	1.01	1.58
	Often	38	29.2	63	24.8	16	13.1		0.25	1.12	0.91	1.38
	Always	31	23.8	69	27.2	38	31.1			Ref.		
72. Reduced working hours during outbreaks	Never	38	29.2	88	34.6	45	36.9	0000	0.21	0.85	0.66	1.09
	Sometimes	48	36.9	73	28.7	14	11.5		0.01	0.72	0.57	0.93
	Often	30	23.1	48	18.9	24	19.7		0.28	0.87	0.68	1.11
	Always	14	10.8	45	17.7	39	32.0			Ref.		

* Multivariate Model 1s controlled for gender, age, marital status, place of residence, occupation, place of work, income level, first time working with COVID-19 patient, receive training to deal with COVID-19 patients, receive training to treat COVID-19 patients, work with COVID-19 patients in treatment centers, coping factors. Sig. = significance. ** Multivariate Model 2 controlled for gender, age, marital status, place of residence, occupation, place of work, income level, first time working with COVID-19 patient, receive training to deal with COVID-19 patients, receive training to treat COVID-19 patients, work with COVID-19 patients in treatment centers, and motivation factors. Sig. = significance.

**Table 4:** Correlation between stress scoring, coping scoring, and motivation scoring, and their association with the stress index (N = 506).

	Mean	Std. Deviation	Correlation Coefficient with Stress Scoring		Adjusted Analysis * with Stress Index			
			Spearman’s rho	Significance	Sig.			
Coping scoring	23.3	6.86	0.25	0.000	0.000	1.06	1.03	1.09 ^†^
Motivation scoring	14.8	8.19	0.067	0.14	0.078	1.02	0.99	1.04 ^‡^

* Multivariate Models controlled for gender, age, marital status, place of residence, occupation, place of work, income level, first time working with COVID-19 patient, receive training to deal with COVID-19 patients, receive training to treat COVID-19 patients, work with COVID-19 patients in treatment centers, coping scoring ^†^, and motivation scoring ^‡^. Sig. = significance.

## Data Availability

The data sets used and/or analyzed during the current study are available from the corresponding author on reasonable request.

## Abbreviations

COVID-19	Coronavirus disease 2019
HCWs	Healthcare workers
WHO	World Health Organization
